# Notes and News

**Published:** 1905-02-04

**Authors:** 


### Notes and News.

The King, as patron of King Edward VII.'s Hospital for Officers, has approved the appointment, by the Prince of Wales as president, of the following additions to the list of the honorary medical staff:—To be Honorary Surgeons and Physicians, Mr. Watson Cheyne, M.B, C M. Edin., F.R.C.S., F.R.S., Mr. Pearce Gould, M.S., F.R.C.S., and Dr. Bruce Porter.

The Queen has promised to open the bazaar which is to be held in aid of the funds of Westminster Hospital on May 23. The Prince of Wales will perform a similar ceremony on May 24, and the Duke of Connaught on May 25.

The Duchess of Albany, as president of the Ladies' Association of the Royal Waterloo Hospital for Children and Women, has fixed Tuesday, February 28, at three o'clock, for a meeting of the members of the association and their friends to be held at the hospital, Waterloo Bridge Road. The Archbishop of Canterbury will be one of the speakers.

Prince Eitel Friedrich, second son of the German Emperor, is suffering from a severe attack of inflammation of the lungs, complicated by pleurisy.

Amongst the latest contributions received at the Bank of England for King Edward's Hospital Fund for London are the following:—Annual subscriptions—Messrs. Elkington and Co., Limited, £10 10s.; Mr. H. Eschevege, £10 10s.; General G. E. Baynes, £10. Donations—Lieutenant Marston, R.N., £25; Sir Richard Couch, £20.

The Council of King's College, with a view to preparing the way for the removal of King's College Hospital to Camberwell, and the separation of preliminary and intermediate medical studies, have dissolved the Medical Board, and created a Board for advanced medical studies, which will superintend the teaching work of the hospital, and be organised as a separate department. The preliminary and intermediate studies have been transferred to the Faculty of Science under a separate Board.

Surgeon-General W. J. Fawcett, M.B., C.B., has been appointed deputy director-general of the Army Medical Service, in succession to Surgeon-General Keogh, now director-general.

The Cross of the Legion of Honour has been conferred upon Dr. George Ogilvie, senior physician to the French Hospital in London, in recognition of his devoted services to that institution.

The bacteriologists of New York have presented a report confirming the statement that the 14 deaths which occurred on board the Red Star liner *Vaderland*, were due to pneumonia. After a thorough disinfection, the *Vaderland* has been released from quarantine.

Among those who have accepted the invitation of the President and Vice-President of the Royal College of Surgeons to dine at the college on the occasion of the Hunterian Festival on Feb. 14, are the Duke of Abercorn, Lord Onslow, the Bishop of Oxford, Lord Cheylesmore, Sir James Bryce, M.P., Sir Herbert Maxwell, M.P., Sir William Broadbent, Sir William Ramsay, and Sir Frederick Treves.

At the invitation of Colonel and Mrs. Barrington Foote a drawing-room meeting to promote the objects of the League of Mercy in the Brentford district was held last week at Kneller Hall, Whitton, Hounslow. Mr. Montague Sharpe was in the chair, and there was a large attendance. Mr. Herbert J. Paterson, of the East Marylebone district, gave an address and was followed by Mrs. R. L. Harrison, who told how the organisation of the League was carried on in Fulham, which last year contributed upwards of £1,000.

Mr. D. J. Mackintosh, medical superintendent of Glasgow Western Infirmary, will read a paper on "The Control of Hospital Expenditure with Efficiency," at a meeting of the Hospitals Association on Friday, March 3. Tickets for admission to the meeting, which will be held in the hall of Charing Cross Hospital, by permission of the council of the hospital, can be obtained on application to the hon. secretary of the Hospitals Association, 28 Southampton Street, Strand, W.C.

On Monday Professor Boyce, of Liverpool University, Dr. Evans, and Dr. Clarke, members of the 13th expedition of the Liverpool School of Tropical Medicine, were entertained at luncheon in Liverpool, after their recent return from the West Coast of Africa. Professor Boyce, in the course of an interesting speech, said that the expedition directed their attention to health problems at Bathurst, Konakry, and Freetown. In consequence of the vigorous anti-mosquito measures at Bathurst there is a very material improvement in the health of both the Europeans and natives. Great improvement in the health of the people has resulted at Freetown from the measures adopted within the last few years, but the work of sanitation, drainage, and water supply will have to be undertaken. At Konakry, which is under French rule, malaria is, if anything, on the increase, because the old wells in which mosquitos breed have not been shut.

